# Extra Supplement—The Nursing Mirror

**Published:** 1889-07-27

**Authors:** 


*The Hospital*,

July 27, 1889. [Extra Supplement

### The Nursing Mirror.

All Contributions for this Supplement should be addressed to the Editor, The Hospital, 139-140, Salisbury Court, Fleet Street, London, E.C., and should have the word "Nursing" plainly written in left-hand top corner of the envelope

### En Passant.

A WARNING TO MIDWIVES.—A midwife at West Hartlepool lately attended a patient who developed puerperal fever. A medical man was not sent for till a few hours before death occurred. He naturally refused to give a certificate and an inquest had to be held.

THE SURGEON OR LADY-SUPERINTENDENT.—Who ought to have supreme power over the nursing staff—the house surgeon or the lady-superintendent? "The superintendent, of course!" we can hear our readers exclaim, "that was decided long ago by Florence Nightingale." It is strange to learn then that at Norfolk and Norwich Hospital a rule has just been passed which will give the house surgeon power to suspend any nurse. We hope the rule won't stand in force long; Mr. Cadge and Dr. Beverley showed the objections to it clearly but did not speak forcibly enough. We are sure, if the committee considered the subject fully and impartially they would take a more modern and enlightened view.

SICK NURSES AT ADDENBROOKES. — At the quarterly court of governors of Addenbrookes Hospital, the Rev. H. Hall said that £250 had been raised primarily with the view of building a ward for sick nurses. He felt that from the position the hospital took as a training school for nurses, everything should be done that could be done to support the school. Amongst the deficiencies, at the present time, was a place where probationers could be taken care of when ill. They had thirty probationers, young people, some of whom were constantly down with illness. He proposed that a committee be appointed to consider as to a possible site and the probable cost of a ward for sick nurses and probationers, and that the committee report the result of the enquiry to next quarterly court. Dr. Macalister, in seconding, said something should be done. On more than one occasion he had had to attend nurses, and they had either to remain in their own cubicles, or be taken to the wards. That was not desirable for several reasons, among which was one that if sick nurses filled beds in the wards these beds could not be utilised for ordinary patients, for whom they were primarily intended.

THE HOME AT FELIXSTOWE.—The matron of the Suffolk Convalescent Home and Sea-Bathing Infirmary, at Felixstowe, will hold her annual sale of work on the beach on August 30 and 31. Last year this sale produced £90, and if only a few more friends will send contributions the sum will this year run into three figures. An article which sells well and is easy to make is a Nightingale cloak. Two and a half yards of flanelette at fourpence a yard, two yards of lace at a penny, and two yards of ribbon at twopence, and for one shilling and fourpence and half-an-hour's needlework you have a pretty and useful article which will easily sell for half-a-crown. Just now the Felixstowe Home is quite full, and as there are many visitors in the town, it is hoped they will interest themselves both in the home and the sale. Last year 379 patients were admitted. An amusing entry occurs in the men's commonplace book. One poor old convalescent, of sixty or thereabouts, was touchingly effusive, his entry running as follows: "Allow me to return my sincere thanks to the 'matron' (who has proved a mother to me in every sense of the word), and to those connected with the institution, also my fellow-patients." Seeing that the writer was old enough to be the matron's father, the proving a mother to him "in every sense of the word" was a trifle too exuberant.

DARENTH ASYLUM.—The annual inspection took place last week. In the school asylum, of which Miss Wright is the matron, there were seen some 600 children suffering in all phases of weakmindedness. Some were as helpless as infants, while others were seen to be able to work, and, indeed, under constant and skilled care, were shown at work at trades. Some of them proved capable of following mental training, and their capabilities are brought out gradually under a system initiated by Miss Stephens (now Mrs. Dyer), the first head mistress and matron. The visit to the adult asylum showed how vastly the treatment of this class of patients has advanced during the last 20 years. The buildings, while having no architectural pretensions, are not unpleasing to the eye, and are built on the "block" principle. The cost of the adults is only 8d. per diem for food and clothing, and of children a fraction over 6d. The committee and officers were complimented on their self-sacrificing labours on behalf of suffering and helpless humanity.

DISTRICT WORK AT BRIGHTON. — There is a central association at Brighton for nursing the sick poor. At present the association is working with a small staff of nurses, quite inadequate to the great need for their services amongst more than 60,000 poor inhabitants, not reckoning the outlying villages, where similar help is much needed. The nurses, who are thoroughly qualified for their duties by professional training, undertake many cases weekly, some requiring three and four visits daily. These average 600 per annum. They carry out all necessary sanitary arrangements, doing their utmost to comfort and relieve the suffering, instructing the friends and relatives of the patient in all needful ways, and refusing no office of kindness "to those who have none else to care for them." The work is entirely unsectarian, all who need sympathy being helped with equal gladness. At present the nurses are overworked, and Dr. W. J. Stephens has written an appeal for funds to pay for more nurses. He testifies personally to the good work done by the nurses.

THE HAGUE DEACONESSES.—Amongst the papers read at the Women's Congress at Paris was one on the work of deaconesses at Hague, which has been fully reported in the *Women's Penny Paper*. The beginning of the house of the Hague deaconesses was most modest. In the year 1864, three ladies conceived the idea of starting in this town a work similar to the *Ecole Normale* of sick nurses at Lausanne. One of these ladies being owner of a small house, which was admirably suited to this purpose, gave it up to the committee for the space of four years, and they asked Mademoiselle de Bronoro to take charge of the direction. She accepted the offer, not without hesitatation. Before beginning her task, she first visited similar establishments in Lausanne and Berlin. On her return to the Hague she began her work with five nurses; and it soon became apparent that this institution was much needed. From all parts came demands for the help of the deaconesses to nurse sick people, so that, when the four years' trial was at an end, far from giving up the work, the committee found it necessary to enlarge it. In the report for the year 1888, we see that 337 invalids were taken care of in the house of the deaconesses. At the Hague the deaconesses were required for 193 families, and in other towns for 49 families. Two hundred and fifty-one operations were made in the establishment by different surgeons, and 1,356 invalids were nursed on their way through the town. The number of deaconesses is now 23, and 17 novices. Mdlle. de Bronoro died in 1887, and Mdlle. de Soeterwoude was elected to fill her place.

### The British Nurses' Association.

*(From a Correspondent.)*

"If the objections raised by Dr. Sansom and Miss Lückes are the strongest that can be raised against the British Nurses' Association, the association has little to fear." So said Sir James Crichton Brown at the Mansion House on the 17th; and we are willing, for the sake of argument, to admit the accuracy of his dictum. It is true that he did not touch on the reason given by Dr. Sansom for withdrawing his support and sympathy from the association—his disapproval of its recent tactics. It is true that he spoke of Miss Lückes, one of the most practical women in the nursing profession, as depicting an imaginary examination—a flight of fancy creditable to his own imagination. He left the objections of the "letter-writers and pamphleteers" untouched, save by the verdict we have quoted; but even if we admit that this verdict is just, it by no means follows that the tactics of the British Nurses' Association are justified. Very serious objections to the association and its scheme of registration were brought forward by several speakers at the Mansion House meeting; and among these we may specially name the Lord Mayor, Dr. Matthews Duncan, Sir Henry Acland, and, above all, Sir James Crichton Brown. They were quite unconscious that they were bringing arguments against the association; they believed they were supporting it; but this only enhances the force of their objections. Balaam, the son of Balah, was taken to a mountain top to curse the children of Israel, and was forced against his will to bless them. The gentlemen we have named went to the Mansion House to bless the British Nurses' Association, and against their will, without their knowledge, they have cursed it.

Let us begin with the Lord Mayor's brief but pregnant utterance: "Registration gives no guarantee of personal character, but only of professional training." This is exactly what is felt by the opponents of the present scheme of registration, and is the point on which they base their objection to it. If this register is to do nothing more than is already done by the certificates given by the training schools, is to do rather less than the testimonials given by those who have seen the nurse at her work, what is the good of it to her? And what is the good of it to the public? If a doctor brought a a nurse into a house and said to the family, "This woman spent three years at a hospital, where she was trained in the technique of nursing, and passed certain examinations. I therefore bring her here to nurse a sick member of your family, whose most constant companion she will be at a time when pain and weakness render the patient peculiarly susceptible to outside influences; but, remember, I can give you no guarantee as to her personal character." If a doctor said this, would the head of the family feel quite sure that the nurse was all that he desired? Yet when a register says this, and nothing more, the head of the family is expected to feel this soothing assurance—is, in fact, given to understand that a good nurse has entered the house. Is it possible to conceive the idea of a good nurse without reference to her personal character?

But, indeed, if we are to believe Sir James Crichton Brown, the function of the register proposed by the British Nurses' Association will be "to do nothing in particular, and to do it very well." There will still, he says, be bad nurses as well as good ones; there will still be nurses who fall below the demands of the register as well as nurses who soar above them; to use his own illustration, there will still be the margarine as well as the "best Dorset." So the elimination of the unfit which we have been promised is not to come by means of the register. What, then, is the register to do?

On the other hand, we may point out that the nurse of brilliant attainments and earnest devotion to her work will stand on the same official level as the woman who has taken up nursing simply because she must earn her living in some way. Such a woman does not fail in her examinations, nor show herself lazy during her training; she would gain her place on the register without a doubt, but she certainly would not be an ideal nurse. If the register is only to give a recognised status to mediocrity and to give no proof of the highest competence, it is the best nurses who will have most reason to object to it, for it will make it more difficult for them to obtain the position and recognition they deserve.

But, in truth, we are still left at a loss as to what this much-talked-of registration is to do. There is not to be a central examining board. So Dr. Matthews Duncan assured the meeting on the 17th, adding that if such a board were proposed he would oppose its establishment with all his might. Then is the register simply to be a reprint of the certificates given by the different training-schools? If so, it might be handy as a book of reference, if it were not too bulky, and as such Dr. Bedford Fenwick suggested at a recent meeting that it might be used. But Sir James Crichton Brown will have none of this. He said—and on this point we quite agree with him—that if the head of a family turned up a register and chose a nurse simply by her name and address, he would be very sorry for that head of a family. So would we; we feel as strongly as Sir James Crichton Brown does that some proof of fitness beyond that given by registration is desirable; but therefore we ask all the more curiously, What will be the good of the register?

One thing, it seems, it is destined to accomplish. Dr. Matthews Duncan gave it as his opinion that, at present, trained nurses are too expensive; that, in fact, their terms are quite exclusive for the majority of the middle class. The result of registration would be, he thinks, to bring their services within the reach of all—that is, to lower the price of their services very considerably. We did not know before that nurses were being asked to support a scheme which would reduce their earnings, which are not now very large, to a still smaller sum. If people cannot or will not pay from one to two guineas a week for the entire services of a trained nurse they must be content with the daily visits of a district nurse, who is also trained, which can usually be obtained gratuitously or at very little cost. The great body of nurses should, however, be grateful to Dr. Matthews Duncan for telling them what will be the probable result of the proposed scheme of registration before they have finally committed themselves to it.

We have not touched on the subject of the registration of midwives, of which so much was said at the meeting, much to the surprise of those who have attended the gatherings of the British Nurses' Association, where they probably never heard the word midwife before. It is a subject on which the minds of all people who have given the matter any thought are made up, and it was brought up before the Hospitals' Association, by Dr. Aveling before the British Nurses' Association was established. It has frequently been urged in the columns of THE HOSPITAL. It is one of the things that are sure to come, because it is needed. But the midwife is not parallel with the nurse, but with the male accoucheur; she acts on her own responsibility, not under a doctor's guidance; she is called in to undertake the supervision and management of a certain department of medical science, and all that is demanded of her is proof of the technical knowledge necessary for her work—a thing that registration can guarantee. The nurse's work is technically narrower, morally wider, than the midwife's; they do not stand on the same platform. And this sudden adoption of the midwife, hitherto ignored by the British Nurses' Association, coupled with the disappearance from the association's programme of the much-talked-of Royal Charter, cannot but convey the idea that, conscious that their scheme of registration is collapsing, they are anxious to annex the special work of a much older association—the Midwives' Institute—the managers of which have worked so long and so hard to obtain the registration of midwives. We protest against the British Nurses' Association claiming any credit for a work that was taken up eight years ago, and most fittingly, by the Midwives' Institute. Thanks to the exertions of the latter, notice was given last week of the introduction of a Bill into the House of Commons by Mr. H. Fell Pease, M.P. for the Cleveland Division of the North Riding of Yorkshire. It is heartily to be hoped that it will soon become law, but if it does it will owe nothing to the advocacy of the British Nurses' Association.

It may be urged that the British Nurses' Association is supported by eminent medical men. This is so ; but is this nurses' association supported by eminent authorities on nursing? Every medical man, even if he has attained to special fame in one department or another of his art, is not *ipso facto* competent to speak on nursing. That needs special study as much as obstetrics or ophthalmology or any other branch of medicine. And while on the subjects to which they have given their attention we are only too glad to hear Dr. Matthews Duncan, Mr. Brudenell Carter, and Sir James Crichton Browne, we prefer, on the question of nursing, to listen to those "persons of thought and experience," as Sir Henry Acland admitted they were, whose opinion was expressed in the recently published memorial against the contemplated scheme of registration. They, and not the gentlemen at the Mansion House, are the real authorities on nursing questions.

### Physiology for Nurses.

### No. III. Baths and Their Effects.

The nerves of the skin are arranged in the papillæ, which lie beneath the mucous layer of the skin, and these nerves help to distinguish various sensations ; so that even if you were blindfold or perfectly blind you could at once say whether you were touched with a needle—*i.e.*, pricked—or if your hand was pressed, and whether the instrument with which you were touched was soft or hard, hot or cold, and so on. Tickling is only a slight irritation of the ends of these nerves—some parts of the body are more sensitive to tickling than others ; you can bear far more tickling on the heels and the fingers than you can on the soles of your feet and palms of the hands. It is very remarkable how greatly the sense of touch can be educated and increased by practice. You know what wonderful things blind people can do, how quickly they learn to read with raised type ; other blind people have learned to distinguish various shells by feel, and even to know real from imitation medals. Some parts of the body feel heat and cold more readily than others, and if you want to try the temperature of a bath, for instance, it is not sufficient just to put your hand into it ; if you only trusted to your hand, when you came afterwards to get into the bath you would find it a great deal too hot. The best way is to put your arm into the water, and so judge of the heat of the water. The skin is very useful in helping to give off the extra heat in the body. In health, and when our skin is in proper working order, this goes on without any trouble to ourselves, and we do not notice it. But when we are ill and very feverish, and the skin does not do its duty properly, then we find how unpleasant it is ; then the skin becomes hot and dry, the heat remains in our bodies, we are constantly craving for something to drink, our mouth and throat are so dried up, and we are very miserable. Then the doctor comes and gives something to make the skin perspire and act properly, and we are relieved and feel comfortable again.

Having seen how very important the skin is, and how necessary it is to keep it healthy, if we wish to be healthy ourselves we must now see what means we ought to take to keep it in working order. The easiest way to do this is to keep the skin clean by means of soap-and-water. There is much on the surface of the skin which it is well to remove. There are the dried-up scales, there is the dried sweat and oily matter, and there is the dirt from the air and our surroundings which clings to this oily matter. We should not keep our bodies perfectly clean unless we also used soap, because the oily substances become mixed with the soap, and then the water carries away both the soap, and the dirt, and oily matter. Some people will not wash their faces with soap, but this is very absurd, for the soap does not irritate the skin of the face more than it does other parts of the body. I believe colliers will not wash their backs because they think it will weaken them ; whilst other people bathe their backs or other parts of the body with cold water because they think it strengthens them.

Besides keeping the surface of the skin clean and, therefore, healthy, the next use of cold water is to stimulate it. The first effect of the cold is to drive the blood away from the skin, and as it must go somewhere it is driven inwards to the internal organs, where it gains more heat, and then gradually returns to the skin, bringing with it the extra heat, and hence the glow. Afterwards, if the person is in proper health, the blood returns in larger quantities, and as the skin, as well as every part of us, is nourished by the blood, of course the skin also benefits by it. For those who can stand it, there is nothing to equal a cold bath in the morning ; it gives a feeling of vigour and warmth afterwards, which cannot be otherwise obtained unless it is by active exercise in the open air : it makes the body less liable to chills and to taking cold ; and for those who are unable to take this active exercise, and whose occupation keeps them engaged indoors for many hours every day, the cold bath is all the more needful. With those who lead very active lives, the skin acts well ; a large quantity of perspiration is continually being thrown off by the skin, and at the same time the dry skin and dirt are both carried away, and thus some of the results of the bath are got. Every nurse should try the cold bath, but begin cautiously, and if after a cold bath you feel chilled and depressed and disinclined for work, and your skin remains cold, do not continue it. Many people who cannot stand a perfectly cold bath can take a tepid one and feel great benefit from it, and sometimes you can with benefit gradually accustom yourselves to cold by beginning with tepid water and each day making it a little colder. By this means you accustom the skin gradually to the shock. There are, of course, some people who can never stand a cold bath at all, because their heart or some other organ is too weak to bear the sudden rush of blood sent inwards from the skin. For those who cannot bear a cold bath a towel wrung out in cold water and briskly rubbed over the body has a very good effect and increases the action of the skin to some extent, though not so much as a cold bath. For those who cannot have a cold bath every day, the wet towel is to be strongly recommended, and when there is an opportunity for a bath it will be more easily borne and enjoyed. Sea-bathing and putting salt in the water are also stimulating to the skin, and make it act more freely and increase the blood-supply ; there is no other virtue in the salt beyond this. A cold bath should never be taken too soon after a full meal, because a large supply of blood is required to digest the food, and the withdrawal of the blood from the stomach interferes with digestion, and there is a danger of headache and flatulence following. The best time for a cold bath is on getting up in the morning, when the body is warm, and it should be taken as quickly as possible after leaving bed, and not after the skin has become thoroughly chilled by dawdling about first. In the same way if you are going to a public bath, it is best to get some exercise, so as to be warm when you arrive, but do not go to the other extreme, and plunge into a cold bath after hurrying and getting very hot and breathless.

*(To be continued.)*

### The Princess of Wales and the National Pension Fund.

"The acceptance by the Princess of Wales of the Presidency of the National Pension Fund for Nurses, and the accession of the Prince of Wales as patron, mark a distinct and important stage in the development of that institution. Upwards of 1,000 nurses have now made proposals, and the Fund is therefore established on a permanent basis. It has been objected in certain quarters against the founder of the Fund, Mr. Henry C. Burdett, that he is not a medical man nor a hospital official. Mr. Burdett has, however, as is widely known, spent the greater part of his life in connection with hospitals, in the capacity of medical student at Birmingham and Guy's Hospital for the full curriculum, and as secretary and house-governor, first at the Queen's Hospital, Birmingham, and then at the Seamen,s Hospital, Greenwich, the latter appointment having been held until within the last few years. His untiring interest in all that relates to hospitals and everything connected with them is amply proved by his many published works on hospital questions, his connection with the Hospital Sunday Fund and the Hospitals' Association, and the fact that a medical contemporary not long ago declared him to be the greatest living authority on hospital affairs generally. Nurses and hospital officials for generations to come will have reason to remember Mr. Burdett's name with gratitude, as the founder of the Pension Fund, and as the indefatigable organiser who started it with a cash balance of nearly £30,000."—*British Medical Journal.*

### Everybody's Opinion.

[*Correspondence on all subjects is invited, but we cannot in any way be responsible for the opinions expressed by our correspondents. No communications can be entertained if the name and address of the correspondent is not given, or unless one side of the paper only be written on.*]

### THE PENSION FUND.

Nurse Barbara writes: I beg to express my grateful thanks to those kind gentlemen who have so generously given the £20,000, deposited as security for the National Pension Fund for Nurses, to the bonus fund, and as one of the first thousand desire to thank them for their bountiful gift to form a special bonus fund for our benefit. I cannot find words to convey my gratitude to all the gentlemen working on behalf of the nurses. I think one way of showing how much we appreciate their efforts to help nurses to help themselves would be by contributing towards the bonus fund; if each nurse would give a little, as she can afford, if only a shilling a year, it would be so easy when sending our premiums to put a trifle more to go to the bonus fund.

### REGISTRATION.

Sister Katherine writes: The protest against registration you published last week has come upon your country readers like a thunder-clap. Why, Sir, we have been assured again and again that all the London training schools were in favour of registration, and that is why we nurses in county infirmaries have joined the Association, which has promised to secure a Royal Charter for that object. Please write to me and tell me the meaning of this protest. Remember we are out of the world here and know nothing but what we are told. Of course we are afraid to move now; we don't know what to believe. Please send us a simple statement of the facts and tell us if we ought to withdraw from the B. N. A.

### Presentation.

Mr. J. Acton Southern, late house surgeon of the Derbyshire General Infirmary, was presented by the staff and nurses on Friday, July 19, with a gold watch, as a small acknowledgment of their esteem and regret at his resignation of the office. Mr. Southern has held the appointment for three and a half years.

### Appointments.

[It is requested that successful candidates will send a copy of their applications and testimonials, with date of election, to The Editor, The Lodge, Porchester-square, W.]

Newcastle-on-Tyne Royal Infirmary.—Miss Frances H. Close has been appointed matron and superintendent of nurses to the Newcastle-on-Tyne Royal Infirmary. She is a certificated nurse, matron of the Kensington Infirmary, and late lady superintendent of nurses, Monsall Fever Hospital, Manchester. She was awarded a silver medal by H.M. the Queen, and a bronze star by H.H. the Khedive for services with the troops in Egypt.

Miss Morse, late of Nottingham, has been appointed matron at Harrogate. Miss Fanny Wallace has been appointed matron of the City of Dublin Hospital. Miss Kayes has been appointed matron at Tewkesbury. Miss Katherine Leech has been appointed midwife to the Enfield Charity.

### Vacancies.

The following vacancies are announced:

**Matrons.**

Birmingham Workhouse Infirmary (assistant), £40.

Murray's Royal Asylum, Perth.

**Sisters.**

Bradford Fever Hospital, £30.
Glamorganshire Infirmary.

**Nurses.**

Bentford Union (night).

Birmingham Orthopœdic Hospital.

Brighton Fever Hospital, £28.

British Home for Incurables, £35.

Chelsea Infirmary, £18.

Croydon General Hospital.

Darlington Fever Hospital (night), £20.

Hampshire Nurses' Institute.

Hollond Institution, Nice.

Hospital for Sick Children, E. (three), £25.

Kendal Hospital, £28.

Kent Nursing Institution (several).

Northwood Fever Hospital, £28.

Nurses' Institute, Jersey, £25.

Rivers-street Home, Bath, £25.

Rotherham Hospital (assistant).

Southport Nurses' Home.

Stratford-on-Avon Hospital, £20.

St. William's Hospital, Rochester, £25.

Torquay Sanatorium, £40.

Victoria Home, Bournemouth, £25.

West London Hospital, Hammersmith, £24.

Wolverhampton Victoria Institution.

**District Nurses.**

Moore, Warrington.

Nurses' Institute, Edinburgh.

Regent-street Home, Nottingham.

Rivers-street Home, Bath.

**Probationers.**

Batley Cottage Hospital.

Bradford Infirmary.

Leeds Fever Hospital (two), £16.

Penzance Infirmary.

### Notes and Queries.

To Correspondents.—1. Questions may be written on post-cards. 2. Advertisements in disguise are inadmissible. 3. In answering a query please quote the number. 4. If a private answer is desired, a stamped addressed envelope must be forwarded.

**Queries.**

(48) *Paid Probationers.*—Marion would like to have a list of hospitals where probationers are paid. (Watch our advertisement columns. *Editor.*)

(49) *Books on Nursing.*—Will some kind nurse inform me where I can obtain the following books, what is their price, and which is the most suitable for a probationer? Wallace Anderson's Notes on Nursing; Domville's Handbook for Nurses; Cleland's or Combe's Physiology; also Sir Andrew Clarke's Private Address.

(50) *Nursing in Melbourne.*—Is there any English agent for any Melbourne institution? *Cora.*

(51) *A Trained Nurse* asks: Can any one give me advice as to the best way of obtaining employment on board vessels going to Australia to save passage (or part) money.

(52) *J. F. H. M.* asks: Where could a girl of 15, suffering from paralysis, be received *free* for change of air for a few weeks?

(53) *A. J. R.* asks: Can you tell me where I can obtain water-proofs, specially made for use of friends of patients, to be put on when visiting in the infectious diseases hospital?

(54) *A Constant Reader* asks: Where can you apply to obtain a post as matron in a creche, a place where mothers leave their children by day.

**Answers.**

(43) *Chatelaines.*—You can get one for 3s. 6d. at 48, Mortimer-street. *A. J.*

(49) *Books on Nursing.*—There is nothing better than Domville's Manual, published at 2s. 6d. by Churchill.

(50) *Nursing in Melbourne.*—Apply to Mrs. Mylne, 122, Gloucester-terrace, Hyde Park.

*M. Williams.*—See back numbers of Hospital. Answer 3S, on July 6th, for instance.

*Rowan.*—Read the article "Physiology for Nurses" in the Hospital for July 13th.

